# Eating-Disordered Behavior in Adolescents: Associations with Body Image, Body Composition and Physical Activity

**DOI:** 10.3390/ijerph17186665

**Published:** 2020-09-13

**Authors:** Eliška Štefanová, Peter Bakalár, Tibor Baška

**Affiliations:** 1 Department of Public Health, Jessenius Faculty of Medicine in Martin, Comenius University in Bratislava, 036 01 Martin, Slovakia; eliska.stefanova@gmail.com (E.Š.); tibor.baska@uniba.sk (T.B.); 2 Department of Sports Educology and Humanistics, Faculty of Sports, University of Presov, 080 01 Presov, Slovakia

**Keywords:** eating-disordered behavior, SCOFF questionnaire, adolescents, body dissatisfaction, BMI, body fat percentage, body fat mass, fat free mass, physical activity

## Abstract

Eating disorders (EDs) represent a disparate group of mental health problems that significantly impair physical health or psychosocial functioning. The aim of this study was to present some evidence about the prevalence of eating-disordered behavior (EDB) in adolescents, and explore its associations with body image (BI), body composition (BC) and physical activity (PA) in this age group. Data from 780 adolescents participating in a health behavior in school-aged children (HBSC) study conducted in Slovakia in 2018 were used (mean age 13.5 ± 1.3; 56% boys). Differences in mean values of numerical indicators were evaluated using the independent samples *t*-test. Differences between nominal variables were assessed by the chi-square test. Pearson correlation was used to describe the associations between all the selected variables. EDB was positively screened in 26.7% (208/780) of adolescents, with a higher prevalence in girls (128/344, 37.2%) than in boys (80/436, 18.3%). Significantly higher means of BI, body weight (BW), body mass index (BMI), body fat mass (BFM), body fat percentage (BFP), body fat mass index (BFMI), fat free mass index (FFMI), and SCOFF questionnaire score (SCOFF QS) were found in those positively screened for EDB. Pearson correlation analysis revealed positive associations between EDB and BI, BW, BMI, BFM, BFP and BFMI. The prevalence of EDB is high in Slovak adolescents. Positive associations between EDB, BI, BMI and fat-related body composition parameters support the idea of a more integrated approach in EDs and obesity prevention and treatment. At the same time, gender differences suggest the need for considering gender-specific strategies aimed at girls and boys separately.

## 1. Introduction

Eating disorders (EDs) represent a disparate group of mental health problems characterized by a persistent disturbance in eating or eating-related behavior that results in the altered consumption or absorption of food, and that significantly impairs physical health or psychosocial functioning [1,2]. These behaviors can range from excessive dieting to a full ED syndrome, and they are considered to be the third most prevalent chronic illness among adolescents, after obesity and asthma [3]. Key factors identified in adolescence as predictive for persistent disordered eating in adulthood include weight concerns, body dissatisfaction, excessive concern with weight gain, unhealthy weight control behaviors and dieting, depressive symptoms, food-related parental practises, and disturbed family cohesion, as well as higher exposure to media influences [4,5,6,7,8,9]. Among those, body dissatisfaction (BD) is an aspect of body image (BI) related to negative evaluations of body size, shape, muscularity/muscle tone and weight, and it usually involves a perceived discrepancy between a person’s evaluation of his or her body and his or her ideal body [10]. In general, lower body satisfaction does not serve as a motivator for engaging in healthy weight management behaviors, but rather predicts the use of behaviors that may place adolescents at risk of weight gain and poorer overall health [11]. In this way, poor BI may be a precursor contributing to the development or maintenance of obesity and/or obesity may contribute to poor BI [12]. Additionally, BD represents a potential moderator of the relationship between weight criticism and physical activity (PA) [13]. PA is an important, though understudied, health behavior among persons with features of binge eating disorder and bulimia nervosa [14]. More evidence is available regarding the abnormally high levels of PA in patients diagnosed with anorexia nervosa (AN) [15]. On the other hand, there is a growing body of evidence suggesting that closely monitored, nutritionally supported exercise is safe and may convey multiple benefits in individuals with ED [16,17].

EDs are severe conditions, but little is known about the prevalence or correlates of these disorders from population-based surveys of adolescents [18]. Therefore, the aim of this study is to present some evidence about the prevalence of eating-disordered behavior (EDB) measured by a SCOFF questionnaire designed to be used as a screening tool for EDs, and explore its associations with body image, body composition and physical activity in this age group.

## 2. Methods

### 2.1. Sample and Procedure

Data were obtained from the World Health Organization (WHO) collaborative health behaviour in school-aged children (HBSC) study conducted in 2018 in Slovakia. Three-step sampling was used to obtain a representative sample. Firstly, 140 larger and smaller elementary schools from rural and urban areas were asked to participate. These were randomly selected from a list of all eligible schools in Slovakia obtained from the Slovak Institute of Information and Prognosis for Education. The school response rate (RR) was 77.9%. Secondly, data from 8405 adolescents from the 5th to 9th grades of elementary schools in Slovakia were obtained using the HBSC survey (mean age 13.4 ± 1.3; 50.9% boys). Thirdly, 10% of participating elementary schools were randomly selected for anthropometric measurements, with 888 adolescents being measured. The data-cleaning phase led to the exclusion of 108 adolescents (12.2%) and to a final study sample of 780 adolescents aged 11 to 15 years (mean age 13.5 ± 1.3; 56% boys).

#### Ethical Statement

The study was approved by the Ethics Committee of the Medical Faculty of the Pavol Jozef Safarik University in Kosice (approval code: 16N/2017). Parents were informed about the study via the school administration and could opt out if they disagreed with their child’s participation. Participation in the study was fully voluntary with no explicit incentives provided for participation.

### 2.2. Variables

The selected variables for analyses consist of HBSC questionnaire items (EDB, BI, PA), body mass index (BMI) based on objectively measured body height (BH) and body weight (BW) and body composition (BC) parameters measured by the bioimpedance method (body fat mass (BFM), body fat percentage (BFP), fat free mass (FFM), skeletal muscle mass (SMM), minerals (Min), proteins (Pro)).

#### 2.2.1. Eating-Disordered Behavior

EDB was assessed by the SCOFF questionnaire, an instrument originally designed by Morgan et al. [19] to be used routinely as a screening tool for EDs. The SCOFF questionnaire has been validated in several studies in different settings and with different populations of adolescents and young adults, and showed good sensitivity and specificity [4,20,21,22]. The items of the questionnaire and its response categories are as follows:

- Q1: Do you believe yourself to be fat even when others say you are too thin? (Yes/No; body image distortion)
- Q2: Do you worry you have lost control over how much you eat? (Yes/No; loss of control over eating)
- Q3: Would you say that food dominates your life? (Yes/No; high impact of food on life)
- Q4: Have you recently lost more than one stone in a three-month period? (Yes/No; weight loss)
- Q5: Do you make yourself sick because you feel uncomfortably full? (Yes/No; deliberate vomiting)

The item “weight loss” was reformulated because of the fact that the weight unit “stone” is not used in Slovakia. Weight loss was therefore defined as loss of more than 6 kg in 3 months [4]. As suggested in other studies, a positive screening status was defined as ≥2 positive answers [4,19,23]. Although the negative predictive value (the proportion of those with negative screening results, who did not actually have an ED) is very high, the positive predictive value is low; that is, the SCOFF has a tendency towards overinclusion [4,20,24]. The SCOFF questionnaire score (SCOFF QS) was calculated by counting each “yes” answer within the questionnaire.

#### 2.2.2. Body Image

The BI item has been included in the HBSC study since the 1993/94 survey and developed internally for HBSC study use. Similar kinds of questions have been used in several other health-related questionnaires of proven validity [25]. The test–retest stability was found to be excellent (ICC = 0.81; 95% CI = 0.76–0.85) in one Finnish study [26]. The BI item measures BD related to self-perceived body weight. This dimension of BI has particular importance, as subjective wellbeing and weight-reduction behavior are highly associated with it. Body-weight satisfaction may change markedly during adolescence (especially in puberty) due to quick and significant somatic changes, so it may have an impact on mental wellbeing and behavior [27]. The item and its response categories are as follows:

*Do you think your body is...?* (much too thin; a bit too thin; about the right size; a bit too fat; much too fat)

For the purposes of statistical analyses, numerical values were set for each of the response categories as follows: 1—much too thin; 2—a bit too thin; 3—about the right size; 4—a bit too fat; 5—much too fat.

#### 2.2.3. Body Composition

The measurement of BC was realized by the bioimpedance analysis method (BIA) using InBody 230 device (Biospace Co., Ltd., Seoul, Korea) following the manufacturer’s measurement instructions [28]. This BIA device has been shown to be reasonably accurate for prevalence studies in adolescents [29].

Before the measurement itself, the BH was measured using Anthropometer A-226 (TRYSTOM Co., Ltd., Olomouc, Czechia) following the measurement instructions [30]: adolescents were asked to take an upright position touching the wall with their heels, buttocks and shoulders, with the head oriented in the so-called Frankfurt horizontal plane.

The BW, BFM, FFM, SMM, Min and Pro measurements and calculations of BMI and BFP were done simultaneously during BIA. Before the measurements were made, the adolescents were instructed to dress in a maximum of a t-shirt and trousers or a skirt. The starting weight was set at −0.5 kg, in order to take into account that we were not weighing the adolescents in underwear [31]. Body fat mass index (BFMI) (kg/height (m)^2^) and fat free mass index (FFMI) (kg/height (m)^2^) were calculated separately using previously measured data [32]. The prevalence of extreme underweight, underweight, overweight and obese adolescents was determined using BMI *z*-scores for Slovak children and adolescents [33]. Overweight and obesity were defined as BMI ≥ 90th and ≥ 97th age- and sex-adjusted percentiles in adolescents. Extreme underweight and underweight were defined as BMI ≤ 3rd and ≤10th age- and sex-adjusted percentiles in adolescents.

#### 2.2.4. Physical Activity

PA was measured by an item adapted for use in the HBSC study from the item developed by Prochaska et al. [34] for the purposes of clinical practice with adolescents. The authors validated it against seven-day continuous measurement using an accelerometer (*r* = 0.40, *p* < 0.001) and observed its substantial test–retest stability (intraclass correlation coefficient (ICC) = 0.77). The item and its response categories are as follows:

Over the past 7 days, on how many days were you physically active for a total of at least 60 min per day? Please add up all the time you spent in physical activity each day (0 days; 1 day; 2 days; 3 days; 4 days; 5 days; 6 days; 7 days).

### 2.3. Statistical Analysis

Differences in mean values of numerical indicators were evaluated using the independent samples *t*-test. Differences between nominal variables were assessed by the chi-square test. Pearson correlation was used to describe the associations between all selected variables.

The data were analyzed in IBM SPSS version 21.0 (IBM, New York, NY, USA).

## 3. Results

### 3.1. Epidemiological Data of the Sample

EDB was positively screened in 26.7% (208/780) of adolescents, with higher prevalence in girls (128/344, 37.2%) than in boys (80/436, 18.3%). Girls had a significantly higher SCOFF QS than boys (mean 1.29 ± 1.00 and 0.89 ± 0.90 respectively; *p* < 0.001). Girls were significantly more represented within the positive answers of the SCOFF questionnaire items Q1 (body image distortion; *p* < 0.001), Q2 (loss of control over eating; *p* < 0.001), and Q5 (deliberate vomiting; *p* < 0.05). Boys were significantly more represented within the item Q4 (weight loss, *p* < 0.05). Gender difference in item Q3 (high impact of food in life) was non-significant with a slightly higher representation of boys (Table 1).

Overall BD (adolescents thinking that their body is a bit too fat or much too fat) was 24.5% (191/780) with higher prevalence in girls (104/344, 30.2%) than in boys (87/436, 20%). Not meeting the WHO global recommendations on PA was reported by 75.4% of adolescents, with higher prevalence in girls (286/344, 83.1%) than in boys (302/436, 69.3%). The prevalence of underweight boys was 6.7% (29/436), of those 31% (9/29) were extremely underweight. In girls, 6.1% (21/344) were underweight and of those 28.6% (6/21) were extremely underweight. Of the boys, 21.1% (92/436) were overweight, with 28% (35/92) of them being classified with obesity. In girls, 17.7% (61/344) were overweight, with 36.1% (22/61) of them being classified as obese.

### 3.2. Differences between EDB Negatively and Positively Screened Groups of Adolescents

Statistically significant differences between the groups that were negatively and positively screened for EDB were found in BW (*p* < 0.001), BMI (*p* < 0.001), BFM (*p* < 0.001), BFP (*p* < 0.001), BFMI (*p* < 0.001), FFMI (*p* < 0.05), BI (*p* < 0.001) and SCOFF QS (*p* < 0.001), with higher means in those positively screened. These differences were observed in boys and girls separately as well. In boys, they were complemented by a difference in PA (*p* < 0.05). In girls, they were complemented by differences in FFM (*p* < 0.001), SMM (*p* < 0.001), Min (*p* < 0.001) and Pro (*p* < 0.001) (Table 2).

### 3.3. Correlation Analysis

Pearson correlation analysis revealed positive associations between SCOFF QS and BI (*p* < 0.001), BW (*p* < 0.001), BMI (*p* < 0.001), BFM (*p* < 0.001), BFP (*p* < 0.001) and BFMI (*p* < 0.001). PA was positively associated with SCOFF QS only in boys (*p* < 0.05) and FMM (*p* < 0.001), FFMI (*p* < 0.001), SMM (*p* < 0.001), Min, Pro only in girls (Table 3).

## 4. Discussion

We searched for prevalence of EDB and its correlates in the sample of 780 adolescents aged 11 to 15 years in Slovakia. The overall prevalence of EDB assessed by SCOFF questionnaire was higher in our sample than in comparable samples of German (19.3%), Finnish (20%), Austrian (22.8%) or Spanish (23.2%) adolescents [4,35,36,37]. The high prevalence of EDB in this population group in Slovakia should be of great concern given that EDB is associated with a wide range of psychopathological and psychosocial problems [24].

We did observe twofold gender differences in the prevalence of EDB, which is a consistent finding throughout the literature [4,35,36,37]. This can be attributed to a higher prevalence of some particular EDs in girls than boys; for example, girls answered positively twofold more often on SCOFF questions Q1, Q2 and Q5. Hautala et al. [35] found similar patterns in Q1 and Q2, but unlike in our sample, they did not find other significant differences. Despite these differences, much of the research on gender differences emphasizes the similarities between males and females with EDs and endorses the continuation in using similar strategies to detect and treat EDs in both genders [38]. Our findings suggest that ED prevention and treatment programmes may need to consider gender-specific strategies aimed at girls and boys separately [39].

BI differences between EDB negatively and positively screened in our sample of adolescents are in line with previous studies, which have demonstrated that body image disturbance is not only a symptom of EDs, but can also be considered as a risk factor for their development [40]. Our finding about the positive association between SCOFF QS and BI supports that conclusion. The SCOFF QS increases with a higher perception of the body as fat. The positive associations of SCOFF QS, BMI, BFM, BFP and BFMI complement this finding on the body composition level. In our sample, these parameters were significantly higher in EDB positively screened adolescents, both boys and girls. Regarding the BMI, BFM and BFP, the results are in line with previous studies showing that BMI, waist circumference and BFM are positively related with the risk of eating disorders [41,42,43]. Clinicians therefore should be aware that as their clients’ BMI and BFP increase, BI changes by perceiving the body as fatter, and the risk of EDB increases [12]. Consequently, significantly higher values of FFMI in the total sample and both boys and girls reflect a positive and linear relationship between FFM and BFM [44]. This relationship was, in girls, accompanied by significantly higher values of FFM, SMM, Min and Pro.

PA was significantly higher only in positively screened boys compared to negatively screened boys. It may be attributed to our finding about a higher frequency of boys reporting to have recently lost 6 kg in a three-month period. Losing weight together with excessive levels of PA are characteristic for a subgroup of individuals with AN [2]. Since the SCOFF questionnaire itself does not allow for exact cross-sectional ED classification in adolescents at the moment, future research should perhaps focus on the development of a similar clinical algorithm as the recently developed Expali^TM^ [45,46]. This can provide more comprehensive knowledge about prevalence rates of eating and other mental disorders, which is central to the provision of health care in the community [4].

The major strength of this study relates to its large sample of adolescents with objectively measured body composition, allowing for more body composition variables to be analyzed within the context of EDB. As a result, the study presented evidence about differences between EDB positively and negatively screened adolescents and selected associations. However, some limitations must be mentioned. Firstly, boys were slightly more represented in the sample than girls, which could affect the results. Secondly, the cross-sectional design of the study does not allow causal relationships to be drawn from its results. Thirdly, only self-reported measurements were used to measure EDB, which could have led to reporting bias, particularly in participants with ego-syntonic EDs such as AN [47]. Furthermore, disordered eating in males may be associated with symptoms that are not assessed by the SCOFF questionnaire, e.g., the desire to gain muscle (as opposed to the desire to be thinner) [47,48].

## 5. Conclusions

There is a high prevalence of EDB in Slovak adolescents. Positively screened adolescents have a worse profile compared to their negatively screened peers in BI, BC and PA (in boys). The BI item has significantly higher values in those EDB positively screened, which indicates that they perceive their body as fat. This is accompanied by significantly higher values of BW, BFM, BFP, BFMI, FFMI and overall SCOFF QS. Frequency of PA was significantly higher only in boys, which may indicate use of PA in an unhealthy way. Positive associations between EDB, BI, BMI and fat-related body composition parameters suggest that there is an overlap in these conditions and supports the idea of a more integrated approach in EDs and obesity prevention and treatment [1]. At the same time, gender differences suggest the need for considering gender-specific strategies aimed at girls and boys separately.

## Figures and Tables

**Table 1:** Differences between boys and girls in SCOFF items and score.

	Total *n*/% of Yes Answers	Boys (*n*/% within Yes Answers and % within Gender)	Girls (*n*/% within Yes Answers and % within Gender)
Q1	192/24.6%	68/35.4% (15.8%)	124/64.6% (36.5%) **
Q2	158/20.3%	51/32.3% (11.8%)	107/67.7% (31.2) **
Q3	351/45%	189/53.8% (43.6%)	162/46.2% (47.1%) ^ns^
Q4	103/13.2%	69/67% (15.9%)	34/33% (9.9%) *
Q5	31/4%	11/35.5% (2.5%)	20/64.5% (5.8%) *
SCOFF QS	-	0.89 ± 0.90	1.29 ± 1.00 **

Note: **—*p* < 0.001; *—*p* < 0.05; ^ns^—non significant; Q1—body image distortion; Q2—loss of control over eating; Q3—high impact of food on life; Q4—weight loss; Q5—deliberate vomiting; SCOFF QS—SCOFF questionnaire score (number of “yes” answers).

**Table 2:** Differences between eating-disordered behavior (EDB) negatively and positively screened groups of adolescents in selected variables.

Variable	Total (*n* = 780)		Boys (*n* = 436)		Girls (*n* = 344)	
	Negatively Screened (*n* = 572)	Positively Screened (*n* = 208)	Negatively Screened (*n* = 356)	Positively Screened (*n* = 80)	Negatively Screened (*n* = 216)	Positively Screened (*n* = 128)
Age	13.46 ± 1.31	13.41 ± 1.24	13.53 ± 1.30	13.30 ± 1.25	13.34 ± 1.32	13.49 ± 1.24
Body height (cm)	162.38 ± 10.74	162.08 ± 9.03	163.98 ± 11.84	164.06 ± 10.75	159.74 ± 7.96	16.85 ± 7.54
Body weight (kg)	51.33 ± 12.19	57.47 ± 13.31 **	52.94 ± 13.20	60.87 ± 17.01 **	48.68 ± 9.80	55.34 ± 9.84 **
BMI	19.26 ± 3.19	21.71 ± 3.97 **	19.44 ± 3.27	22.31 ± 4.75 **	19.00 ± 3.04	21.34 ± 3.35 **
Body fat mass (kg)	9.61 ± 5.98	15.23 ± 8.23 **	8.79 ± 6.00	15.00 ± 10.09 **	10.98 ± 5.7	15.37 ± 6.85 **
Body fat percentage (%)	18.13 ± 8.35	25.39 ± 9.47 **	16.02 ± 8.07	23.08 ± 11.08 **	21.60 ± 7.65	26.83 ± 8.03 **
Body fat mass index	3.66 ± 2.27	5.8 ± 3.11 **	3.29 ± 2.24	5.56 ± 3.68 **	4.28 ± 2.20	5.96 ± 2.69 **
Fat free mass (kg)	41.74 ± 9.62	42.24 ± 8.67	44.16 ± 10.55	45.86 ± 11.29	37.70 ± 6.05	39.97 ± 5.45 **
Fat free mass index	15.59 ± 1.93	15.91 ± 1.81 *	16.15 ± 1.99	16.76 ± 2.27 *	14.69 ± 1.44	15.39 ± 1.21 **
Skeletal muscle mass (kg)	22.74 ± 5.79	23.02 ± 5.21	24.23 ± 6.34	25.22 ± 6.81	20.28 ± 3.63	21.64 ± 3.24 **
Minerals (kg)	2.89 ± 0.66	2.97 ± 0.60	3.03 ± 0.74	3.19 ± 0.78	2.67 ± 0.44	2.85 ± 0.41 **
Proteins (kg)	8.20 ± 1.92	8.29 ± 1.73	8.70±2.10	9.02 ± 2.26	7.39 ± 1.20	7.84 ± 1.08 **
Body image	2.82 ± 0.81	3.46 ± 0.87 **	2.81 ± 0.79	3.3 ± 0.93 **	2.85 ± 0.84	3.56 ± 0.82 **
SCOFF QS	0.58 ± 0.49	2.39 ± 0.64 **	0.55 ± 0.50	2.39 ± 0.70 **	0.64 ± 0.48	2.39 ± 0.60 **
Physical activity (days)	5.44 ± 1.97	5.40 ± 1.97	5.62 ± 1.95	6.09 ± 1.90 *	5.14 ± 1.97	4.97 ± 1.89

Note: **—*p* ˂ 0.001; *—*p* ˂ 0.05; BMI—body mass index; SCOFF QS—SCOFF questionnaire score (number of “yes” answers).

**Table 3:** Correlations between SCOFF Questionnaire Score and selected variables.

		BI	BH	BW	BMI	BFM	BFP	BFMI	FFM	FFMI	SMM	Min	Pro	PA
SCOFF QS	Total (*n* = 780)	0.280 **	ns	0.194 **	0.264 **	0.307 **	0.298 **	0.304 **	ns	ns	ns	ns	ns	ns
	Boys (*n* = 436)	0.189 **	ns	0.191 **	0.247 **	0.280 **	0.237 **	0.271 **	ns	ns	ns	ns	ns	0.120 *
	Girls (*n* = 344)	0.342 **	ns	0.299 **	0.311 **	0.284 **	0.267 **	0.274 **	0.208 **	0.254 **	0.209 **	0.215 **	0.210 **	ns

Note: **—*p* < 0.001; *—*p* < 0.05; ns—non significant; SCOFF QS—SCOFF questionnaire score (number of “yes” answers); BI—body image; BH—body height (cm); BW—body weight (kg); BMI—body mass index; BFM—body fat mass (kg); BFP—body fat percentage (%); BFMI—body fat mass index; FFM—fat free mass (kg); FFMI—fat free mass index; SMM—skeletal muscle mass (kg); Min—minerals (kg); Pro—proteins (kg); PA—physical activity (days).
